# Does the Difference in the Aggregation-Sex Pheromone Release Pattern Between *Monochamus alternatus* Hope (Coleoptera: Cerambycidae) and *Monochamus saltuarius* Gebler Ensure Reproductive Isolation in the Cohabitation Area?

**DOI:** 10.1007/s10886-025-01617-y

**Published:** 2025-06-06

**Authors:** Min-Jung Huh, Il-Kwon Park

**Affiliations:** 1 Department of Agriculture, Forestry, and Bioresources, College of Agriculture and Life Sciences, Seoul National University, Seoul, 08826 Republic of Korea; 2 Division of Conservation of Forest Environment, Research Institute of Agriculture and Life Science, College of Agriculture and Life Sciences, Seoul National University, Seoul, 08826 Republic of Korea

**Keywords:** *Monochamus alternatus*, *Monochamus saltuarius*, Monochamol, Aggregation-sex pheromone, Emission pattern difference

## Abstract

In our study, we analyzed diel, daily, and weekly pheromone emission patterns to determine whether pheromone release pattern may facilate reproductive isolation between two species that use the same aggregation-sex pheromone, namely *Monochamus alternatus* Hope (Coleoptera: Cerambycidae) and *Monochamus saltuarius* Gebler, (Coleoptera: Cerambycidae) in areas where they are sympatric. The daily emission of monochamol by males of both species showed irregular but constant patterns, with *M*. *saltuarius* males releasing the pheromone earlier than their *M*. *alternatus* counterparts after cuticular sclerotization. The first emission of the pheromone occurred, on average, 10.6 and 5 days after sclerotization for *M*. *alternatus* and *M*. *saltuarius*, respectively. Weekly patterns of monochamol emission showed a peak in the 3rd week after adult eclosion for both species. *Monochamus saltuarius* released more pheromone than *M*. *alternatus* from 13:00 to 19:00 during three different time periods throughout the day. Both species continuously released pheromones both before and after copulation. *Monochamus saltuarius* displayed mating behavior by mounting and attempting copulation with a glass rod coated with the female extract of *M*. *saltuarius*, while they showed no response to the female extract of *M*. *alternatus*. In contrast, male *M. alternatus* adults did not distinguish conspecifics through substances present on the surface of females. These findings enhance our understanding of the strategies employed to avoid interspecific competition between these two species in cohabitation areas.

**Supplementary Information:**

The online version contains supplementary material available at 10.1007/s10886-025-01617-y.

## Introduction

The global impact of pine wilt disease (PWD), one of the most problematic tree diseases, is a pressing issue that cannot be overstated. It significantly affects the pine forests in Korea, Japan, China, Spain, and Portugal (Ikegami and Jenkins 2018). This disease, caused by the pine wood nematode [PWN, *Bursaphelenchus xylophilus* (Steiner & Buhrer) Nickle], leads to the wilting and death of pine trees by disrupting water movement in the tracheids. In South Korea, the first infected tree was reported on Geumjeong Mt. in Busan in 1988, and the number of affected areas has been steadily increasing since then (Shin 2008).

The male-produced aggregation pheromone of *Monochamus* species was first identified as 2-undecyloxy-1-ethanol (monochamol) from *M. galloprovincialis* (Oliver), a major insect vector of PWN in Europe (Pajares et al. 2010). Monochamol attracted both male and female *M*. *galloprovincialis* when combined with pine volatiles (Pajares et al. 2010). Later, it was discovered that other *Monochamus* species, such as *M. alternatus*, *M. scutellatus* (Say), *M. notatus* (Drury), *M. sutor* L., and *M*. *saltuarius* also released monochamol as their aggregation pheromone (Fierke et al. 2012; Pajares et al. 2013; Teale et al. 2011; Lee et al. 2017). Based on field attraction assays, monochamol demonstrated the properties of an aggregation-sex pheromone, as defined by Cardé (2014), in both *M. alternatus* and *M. saltuarius* (Lee et al. 2017, 2018).

However, since different *Monochamus* species positively respond to the same aggregation pheromone, the chances for mate choice errors and reduced mating success may be high without means of reproductive isolation. Heterospecific mating encounters are frequently associated with loss of fitness (Gröning and Hochkirch 2008). They avoid this competition in various ways, one of which is geographically separating distribution, e.g., *Monochamus carolinensis* Oliver, *M*. *galloprovincialis*, and *M*. *alternatus* have different geographical distributions (Akbulut and Stamps 2012).

Similarly, *M*. *alternatus* and *M*. *saltuarius* had different geographical distributions in South Korea before the PWD outbreak because of the difference in their climatic preferences. *Monochamus alternatus* inhabits warmer areas (Zhao et al. 2008) in the southern parts of South Korea, while *M*. *saltuarius* is distributed in cooler areas in the northern regions of South Korea (Kwon et al. 2006). After the PWD outbreak, *M*. *alternatus* and *M*. *saltuarius* spread their distribution and shared several regions of South Korea such as Gyeonggi, Chungnam, Chungbuk, Jeonbuk, Gyeongnam, Gyeongbuk, and Gangwon province (Han et al. 2023). In the newly established sympatric area, *M. saltuarius* has shifted its host from *Pinus koraiensis*, which was the primary host in its original distribution range, to *P. densiflora* and *P. thunbergii* in regions where *P. koraiensis* is absent. Using the same aggregation-sex pheromone could reduce reproductive success and fitness of both species sharing the same habitat without some mechanisms for reproductive isolation.

Another mechanism that contributes to reproductive isolation in longhorn beetles is species-specific contact sex pheromones. Male beetles recognize conspecific females as potential mates through contact with particular cuticular hydrocarbons present on the female’s body surface (Akutsu and Kuboki 1983; Iwabuchi et al. 1987; Kuwahara et al. 1987; Godinez-Aguilar et al. 2009; Ginzel 2003; Millar and Hanks 2017).

This study explored to determine whether the temporal emission pattern of the aggregation-sex pheromone of *M*. *alternatus* and *M*. *saltuarius* to understand the strategies for avoiding interspecific mating between two species where they overlap in distribution. In addition, we explored the potential existence of a reproductive isolation mechanism involving contact sex pheromones, beyond differences in aggregation-sex pheromone release patterns.

## Materials and methods

### Insects

The *M*. *alternatus* and *M*. *saltuarius* male adults used in the experiment were provided by Osang Kinsect Co., Ltd. (Yesan County, Chungnam Province, Republic of Korea) in pupal form, having been reared under artificial conditions at 28 ± 1 °C and 70% humidity. The pupae were kept at room temperature, and the date of adult emergence was recorded. *Monochamus* adults do not move and remain in the pupal chamber for cuticular sclerotization for about a week after adult eclosion from the pupa (Akbulut and Stamps 2012). The emerged adults were left in the rearing case for one week to allow their cuticles to harden, and then each individual was placed in a separate rearing container with food and distilled water. Current-year branches of *Pinus densiflora* Siebold & Zucc. (for *M*. *alternatus*) and *Pinus koraiensis* Siebold & Zucc. (for *M*. *saltuarius*) were provided as food to allow for sexual maturation.

### Seasonal Occurrence of *M. alternatus* and *M. saltuarius* in the Cohabitation Area

The seasonal flight activity of adult *M*. *alternatus* and *M*. *saltuarius* was investigated using pheromone traps in *P. densiflora* forest stands located in Gyeongju City, Gyeongbuk Province, Republic of Korea, from mid-April to late October 2020. Nine-unit funnel traps (AD Corporation, http://www.2017ad.co.kr/index.html) were hung from rope tied between two trees, with height of the collection cup about 30 cm above ground level. A total of 400 funnel traps were installed at test sites. Collection cups were filled with 300 ~ 400 mL antifreeze solution (main ingredient: ethylene glycol and water, SK Lubricants) to kill and preserve beetles. Lures (AD Corporation) consisted of monochamol, ethanol, and *α*-pinene. Traps were checked three times per month (early, middle, and end), except in April (middle and late), to record the numbers of *M. alternatus* and *M. saltuarius* specimens captured. The exact survey period is provided in Table S1. The collected beetles were transferred to the laboratory, rinsed with water, and identified and counted based on morphological characteristics such as the elytra. A voucher specimen of *M*. *alternatus* and *M. saltuarius* was deposited in the Forest Entomology and Pathology Division, National Institute of Forest Science (Seoul, Republic of Korea).

### Chemicals

Monochamol (purity > 98%), was synthesized according to methods previously described (Lee et al. 2017). Hexane (purity > 98.5%) was purchased from Daejung Chemicals (Siheungsi, Gyeonggi Province, Republic of Korea).

### Collection of Pheromone

**Fig. 1:**
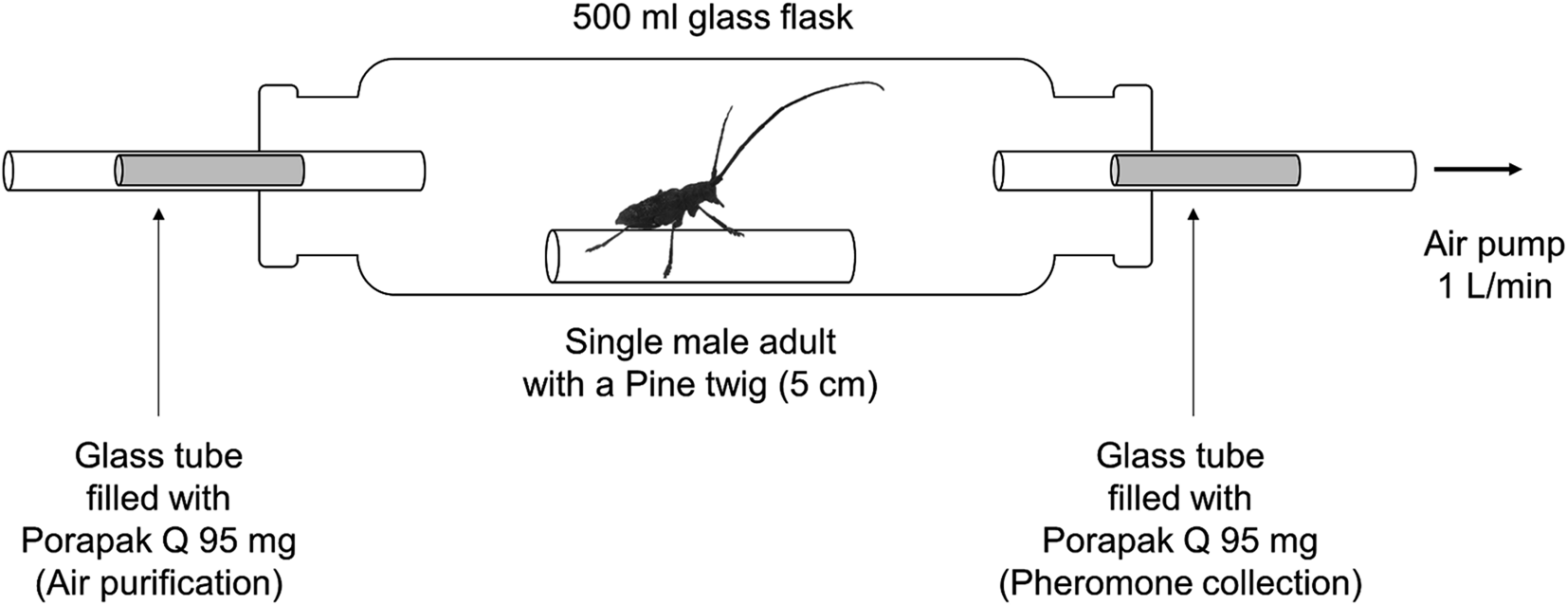
A scheme for collecting aggregation-sex pheromone from a single male adult of *M. alternatus* or *M. saltuarius*

To collect pheromones, fully sclerotized male adults were individually placed into separate glass cylinder (26 cm length, 5 cm o.d.) with inlet and outlet ports for air extraction, and provided with pine branches (Fig. 1). We collected volatiles from 10 *M*. *alternatus* and 12 *M*. *saltuarius*.

Glass tubes (10 cm long, 6 mm o.d., 4 mm i.d.) packed with ca. 95 mg of Porapak Q (Sigma-Aldrich, 80–100 mesh) were inserted at the inlet port for air purification and at the outlet port for aggregation-sex pheromone collection. A glass tube at the cylinder’s outlet was connected to a pump (MV-6005VP, Enomoto Micro Pump., Ltd., Japan) via a rubber hose to generate airflow. The airflow rate was adjusted to 1.0 L/min using an air flowmeter (RMA-21 SSV, Dwyer, USA). Pine branches and cotton wool soaked with water were replaced daily during the experiment. To quantify daily emission amounts of aggregation-sex pheromone, we collected volatiles from male adults for 23 h, excluding one hour for providing water and food and changing the glass cylinder. Pheromones were collected until all individuals of *M*. *alternatus* and *M*. *saltuarius* died.

To determine whether there is a difference in pheromone release between *M*. *alternatus* and *M*. *saltuarius* at different times of the day, we divided the day into three time periods and collected pheromones accordingly. Glass tubes were replaced at 7AM, 1PM, and 7PM at intervals of 12, 6 and 6 h, respectively, to collect pheromones during each time period. Three-week-old *M*. *alternatus* and two-week-old *M*. *saltuarius* were used at their peak pheromone release periods. Nine individuals of each species were used for the time-specific pheromone release measurement over five days.

The amount of pheromone released before and after mating in *M*. *saltuarius* and *M*. *alternatus* was analyzed. Four male adults of each species, which had emerged a month earlier, were used for pheromone collection. The experiment lasted for a total of 10 days, with the glass tube packed with Porapak Q being replaced at the same time each day. On the 4th day of the experiment, male adults were taken out and placed in individual rearing containers with one female adult each, allowing them to mate for two hours. After mating was confirmed, males were returned to the flask, and pheromone was collected again for an additional six days. During the experiment, the glass tubes were replaced daily, and the flasks and food were also replaced with fresh ones.

The volatiles collected in the glass tubes were extracted with 3 ml of n-hexane and then concentrated to approximately 1 µl by evaporating the solvent with nitrogen gas (> 99.999%). The concentrated volatiles were sealed with parafilm and stored in a deep freezer (DTF-35, Nihon Freezer Co., Japan) at -80 °C until analysis.

### Analysis of Pheromone

**Fig. 2:**
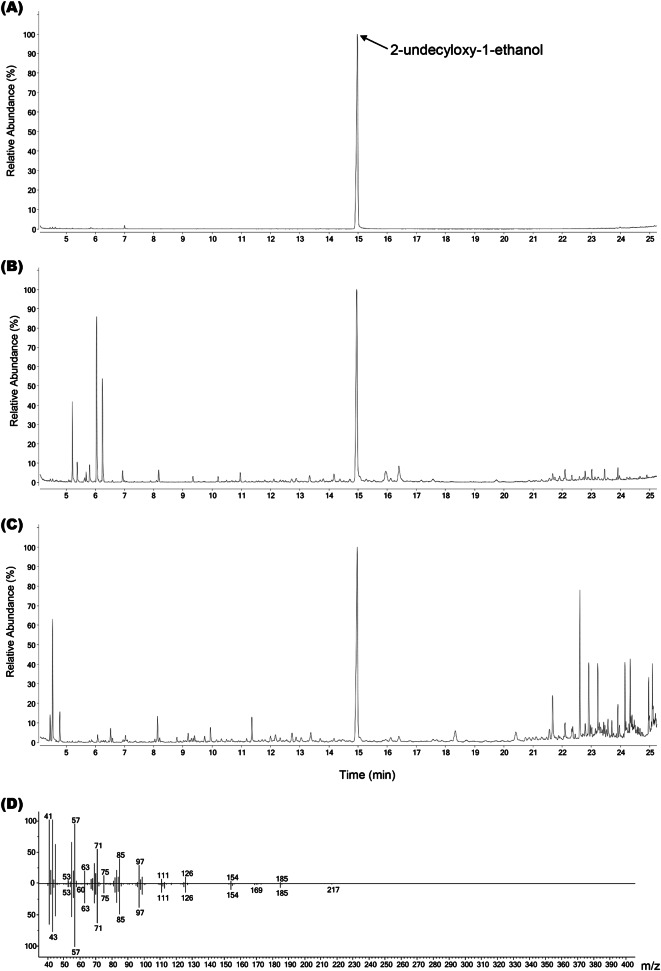
Identification of male-produced sex-aggregation pheromone of *M*. *alternatus* and *M*. *saltuarius* by Gas chromatography-mass spectrometry. 2-Undecyloxy-1-ethanol (standard pheromone) (**A**), 24-h aeration of male *M*. *saltuarius* (27 days old) and *P*. *koraiensis* (**B**), 24-h aeration of male *M*. *alternatus* (35 days old) and *P*. *densiflora* (**C**), EI mass spectra of the male produced aggregation-sex pheromone of *M*. *alternatus* and 2-undecyloxy-1-ethanol (standard pheromone) (**D**)

The collected volatiles were diluted with 200 µl of n-hexane and analyzed using a Gas Chromatography-Mass Spectrometer (GC-MS; GC 7890B, MS 5977B, Agilent Technologies, Santa Clara, CA, USA) to quantify the amount of pheromone emitted daily by adult males throughout their lifetime, during two 6-hour intervals and one 12-hour interval within a 24 -hour period, and before and after mating. The analysis used DB-5MS columns (30 m × 0.25 mm × 1 μm film thickness; Agilent Technologies) with helium as the carrier gas at a 1.0 mL/min flow rate. The oven conditions were as follows: held at 40 °C for 2 min, increased to 95 °C at 20 °C per minute, then to 150 °C at 10 °C per minute and held for 10 min, and finally increased to 300 °C at 30 °C per minute for a total analysis time of 25 min. Standard solutions of monochamol at concentrations of 1, 2, 5, 10, 25, 50, and 100 µg/ml were used to create a calibration curve for quantitative analysis. The selected ion monitoring (SIM) mode was used for quantification analysis to detect trace concentrations. The MS peaks of monochamol in SIM mode were set to m/z values of 43, 45, 57, 63, 71, 85, 97, 111, 126, 154, 171, and 185 (Fig. 2) (Kim et al. 2016).

### Mating Behavior of Male Beetles to the Cuticular Extract of Female Beetles

To investigate the mating behavior of male beetles in response to female cuticular extracts, 20 female *M*. *saltuarius* and *M*. *alternatus* beetles, each 20 days old, were extracted in 200 ml of n-hexane for 10 min. The extracts were concentrated using nitrogen gas. The female extract was then stored in a -80 °C deep freezer until needed for the experiment. To assess the mating behavior of male *M*. *alternatus* and *M*. *saltuarius* in response to the cuticular extracts of female *M*. *alternatus* and *M*. *saltuarius*, 10 µl of the extract, dissolved in 200 µl of n-hexane, was applied to glass rods (0.5 mm in diameter × 20 mm in length) at a concentration of one female equivalent per glass rod (1 FE/rod). A mature male *M*. *alternatus* and *M*. *saltuarius* were then placed near the glass rod and covered with a glass cup (10 cm in diameter × 10 cm in length). The number of male beetles that exhibited mating attempts by firmly holding the glass rod with their forelegs and mandibles while bending their abdomen toward the underside of the bead was recorded (Fig. S1). Each male beetle was used only once in the experiment, which lasted 12 h, from 10 a.m. to 10 p.m. Mating behavior was observed continuously throughout the 12-hour period. Twenty male adults of *M*. *alternatus* and *M*. *saltuarius* were used for each treatment.

### Statistical Analysis

The mean lifespan, mean number of days of pheromone emission within a lifespan, the initial pheromone release time, mean amount of pheromone emission per day, and mean lifetime pheromone emission per individual beetle of *M*. *alternatus* and *M*. *saltuarius* were compared using student’s *t-*test. A generalized linear mixed model (GLMM) with a negative binomial distribution and a log-link function, followed by Tukey’s HSD test as post hoc analysis was used to compare weekly pheromone release amounts. The diel release amounts of pheromone and total amount of pheromone before and after copulation were compared using the paired *t*-test. The daily amount of pheromone emitted by males before and after copulation was compared using a GLMM with a negative binomial distribution and a log-link function, followed by Tukey’s HSD test as a post hoc analysis. The proportion of males displaying mating behavior for female cuticular extract was compared using Fisher’s exact test. All statistical analyses were performed using R Studio (ver. 4.4.3) (R Development Core Team 2013) with ‘dplyr’, ‘glmmTMB’, ‘emmeans’, ‘multcomp’, ‘multcompView’, ‘car’, ‘lawstat’ packages.

## Results

### Seasonal occurrence of *M. alternatus* and *M. saltuarius* in the cohabitation area

**Fig. 3:**
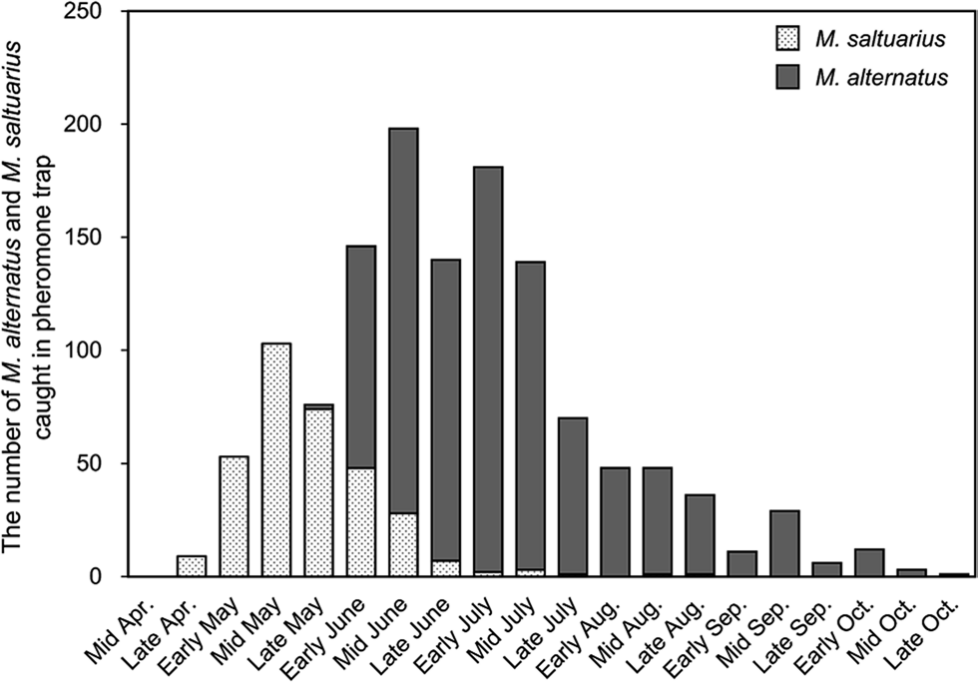
The number of *M*. *alternatus* and *M*. *saltuarius* caught in pheromone traps in the cohabitation area of Gyeongju, Kyungbuck province, Republic of Korea

The first flight of *M*. *saltuarius* was observed in late April, with peak trap catches in mid-May, whereas *M*. *alternatus* flight began in late May and peaked in mid-June and early July (Fig. 3, Table S1) The emergence periods of the two *Monochamus* species overlapped from late May to late August, with the highest number of individuals emerging simultaneously in early to mid-June. Notably, since both species have a lifespan of more than two months, their activity periods are expected to overlap considerably during June and July in areas where they coexist.

### Initial Emission and Amount of Pheromone

The mean adult male lifespan (*t* = 0.2208, *p* = 0.8276), mean number of days during which pheromone emission occurred within a lifespan (*t* = 0.1313, *p* = 0.8968), mean amount of pheromone emission per day (*t* = 0.5777, *p* = 0.5699), and mean lifetime pheromone emission per individual (*t* = 1.1946, *p* = 0.2462) did not differ significantly between *M. alternatus* and *M. saltuarius* (Table 1). However, the initial pheromone release occurred significantly earlier after cuticle hardening for *M*. *saltuarius* males (5.0 ± 2.9 days) than for *M*. *alternatus* males (10.6 ± 4.6 days) (*t* = 3.4683, *p* = 0.0024) (Table 1).

**Table 1:** Mean lifespan, mean number of days of pheromone emission, first emission day, mean and maximum emission amount of pheromone by *M*. *alternatus* and *M*. *saltuarius*

Species	Mean lifespan (day)	Mean No. of days of pheromone emission within a lifespan	Mean No. of days required for the first pheromone emission	Mean amount of pheromone emission (µg/day)	Maximum daily amount of pheromone emission (µg/day)	Mean lifetime pheromone emission per individual (µg)
*M*. *alternatus*	72.8 ± 22.1^1^	37.9 ± 15.1	10.6 ± 4.6	4.6 ± 1.2	30.6	182.1 ± 89.7
*M. saltuarius*	74.8 ± 20.8	36.8 ± 24.0	5.0 ± 2.9*	4.9 ± 1.7	74.9	139.2 ± 78.5
*t* value	0.2208	0.1313	3.4683	0.5777	-	1.1946
*p* value	0.8276	0.8968	0.0024	0.5699	-	0.2462

^1^The number following the mean represents the standard deviation (SD)

^*^Means differed significantly between species (student’s *t-*test, *p* < 0.05)

### Daily and Weekly Emission Pattern of Pheromone

**Fig. 4:**
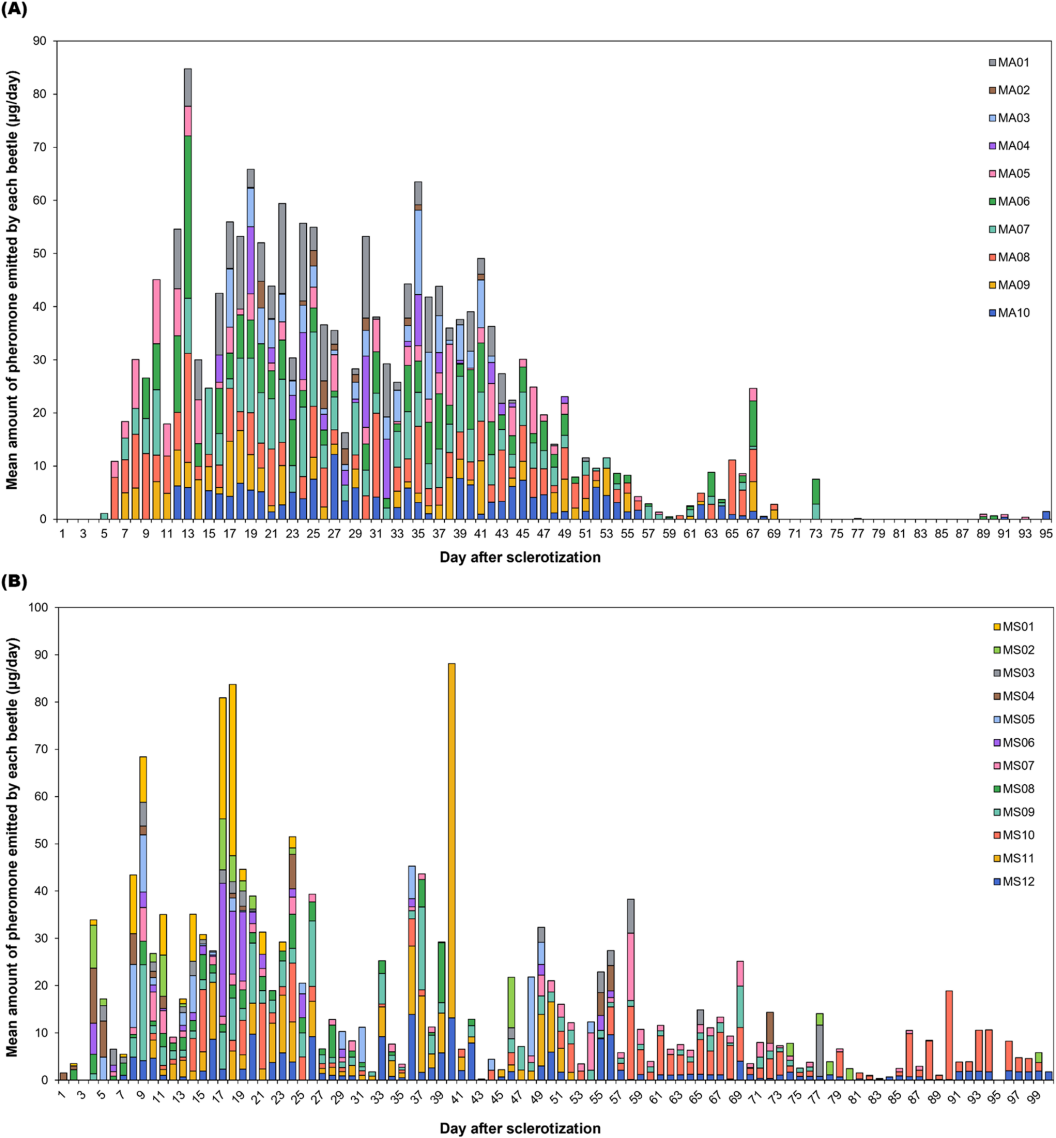
Histogram showing daily pheromone emission by individual male *M*. *alternatus* (**A**) and *M*. *saltuarius* (**B**). Each bar represents the emission profile of male adults over its lifespan, with the length of each colored segment indicating the amount of pheromone released by single male per day. Segments of the same color correspond to the same individual

**Fig. 5:**
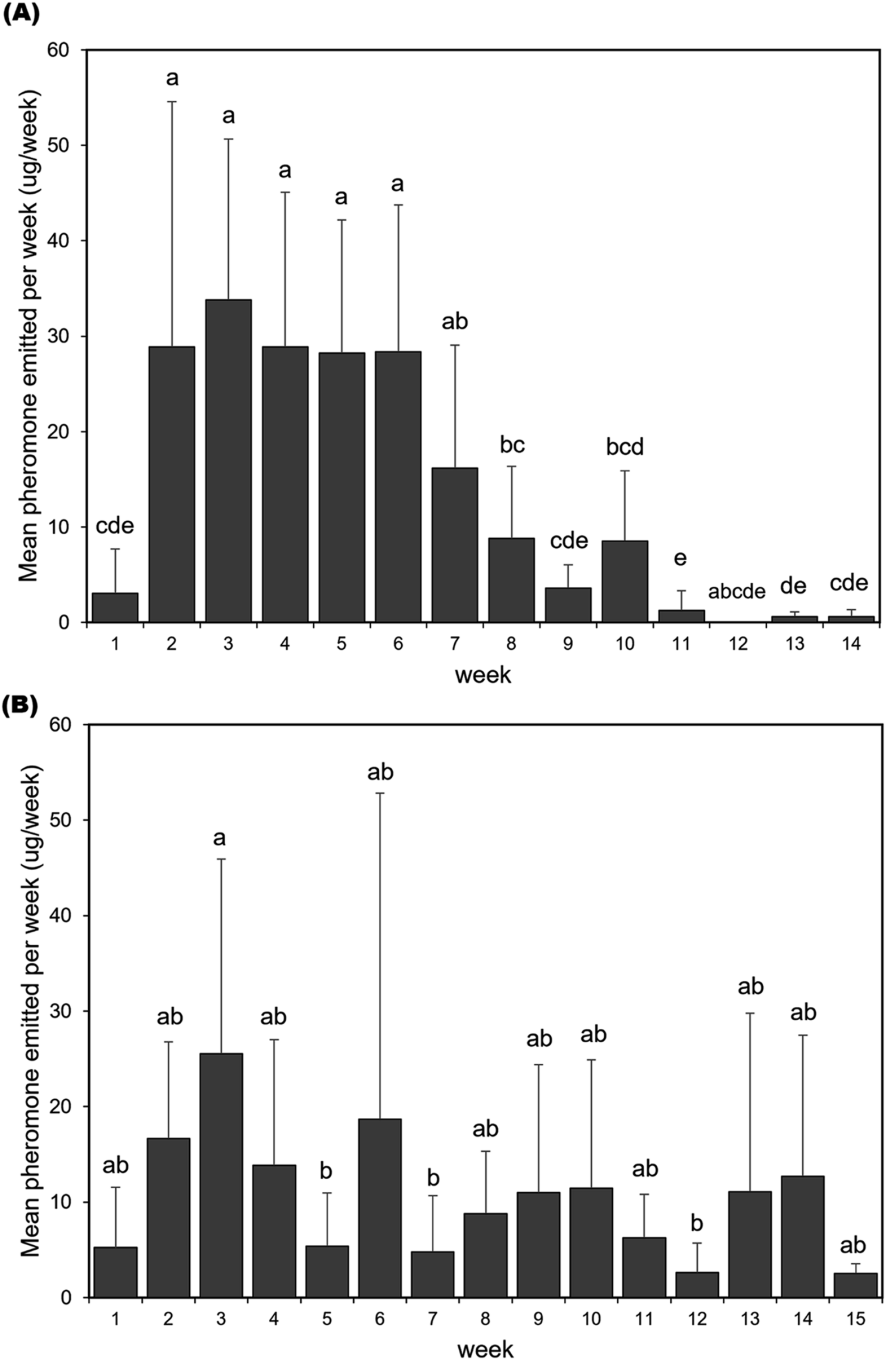
Mean (± SD) weekly amounts of monochamol emitted by *M. alternatus* (**A**) and *M.saltuarius* (**B**). Bars labeled with different letters indicate statistically significant differences among groups, as analyzed by a generalized linear mixed model followed by Tukey’s HSD test

Both *Monochamus* species showed irregular pheromone emission patterns after their initial emissions but consistently emitted pheromones throughout their lives (Fig. 4, Tables S2, S3). *Monochamus alternatus* male adults emitted the high amount of pheromones during the 2nd to 7th weeks after cuticle sclerotization (Fig. 5A; χ² = 106.63, df = 13, *p* < 0.001). However, the amount of pheromone emission decreased starting from the 8th week. *Monochamus saltuarius* male adults generally emitted a consistent amount of pheromones over their lifetime (Fig. 5B; χ² = 31.577, df = 14, *p* = 0.0046).

### Emission Pattern of Pheromone before and after Copulation

**Fig. 6:**
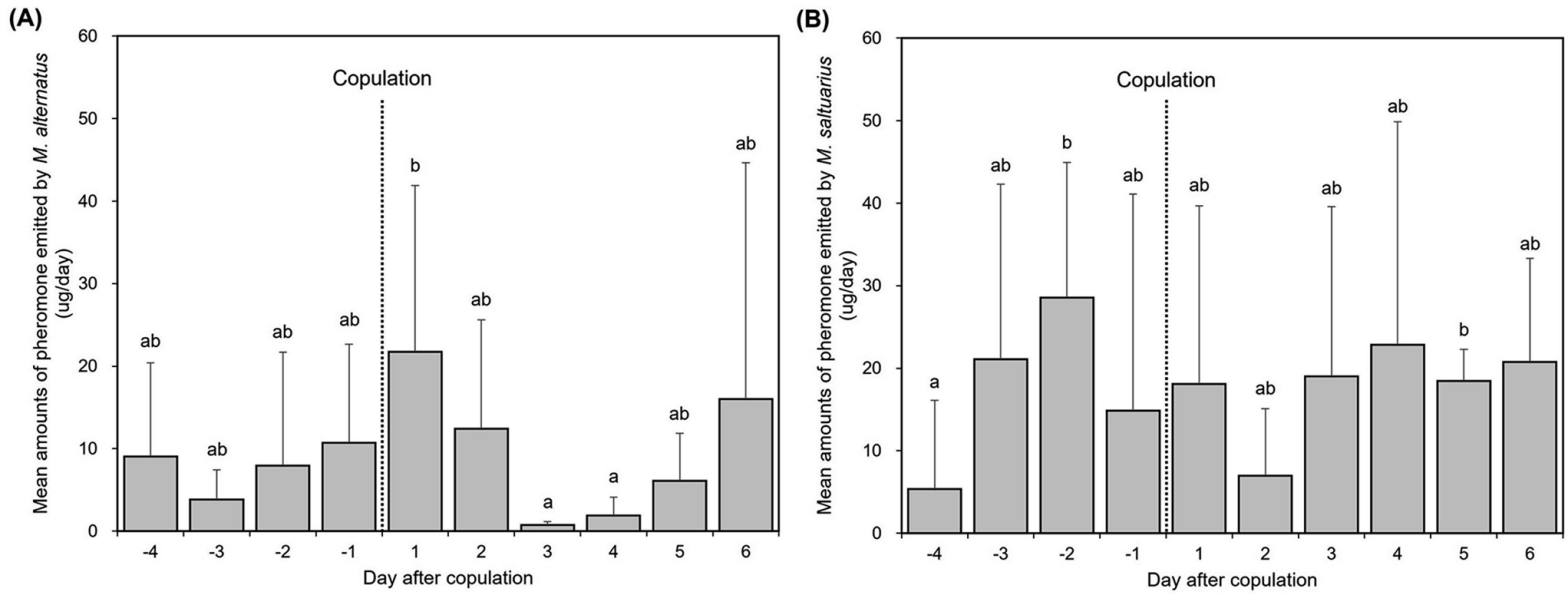
The daily amount (mean ± SD) of monochamol released by *M*. *alternatus* (**A**) and *M*. *saltuarius* (**B**) before and after copulation. Bars labeled with different letters indicate statistically significant differences among groups, as analyzed by a generalized linear mixed model followed by Tukey’s HSD test

Both species released pheromones before and after mating (Fig. 6). There were no significant differences in the total amount of pheromone emitted by *M. alternatus* before and after copulation (*t* = 0.6932, df = 3, *p* = 0.538). There were significant differences in the daily amount of pheromone emission before and after copulation, with the day of copulation showing significantly higher pheromone release than the third and fourth days after copulation (Fig. 6A; χ² = 24.83, df = 9, *p* = 0.0032). However, the amount of pheromone emitted immediately after copulation did not differ significantly from the days before copulation, and showed high variability across days and individuals. *Monochamus saltuarius* also showed no significant differences in total pheromone emission before and after copulation (*t* = 0.0964, df = 3, *p* = 0.9293). However, when comparing daily pheromone emission, *M. saltuarius* males emitted significantly more pheromone two days before and five days after copulation than four days before copulation (Fig. 6B; χ² = 15.574, df = 9, *p* = 0.0763).

### Diel Emission Pattern of Pheromone

**Fig. 7:**
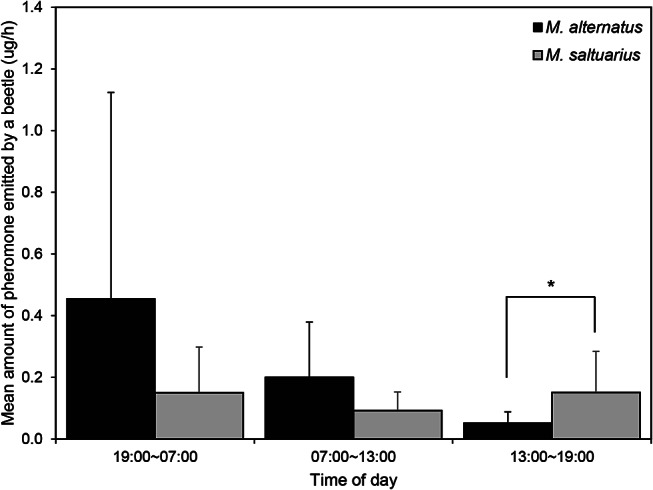
Diel amounts (mean ± SD) of pheromone emitted by two *Monochamus* species. Asterisks indicate significant difference (paired *t*-test, *: *p* < 0.05)

The amount of pheromone emitted during the three time periods within a 24 h period (19:00–07:00, 07:00–13:00, 13:00–19:00) differed significantly between the species during the 13:00–19:00 period only (*t* = 2.16, *p* = 0.0465) (Fig. 7). For *M*. *alternatus* (*n* = 9), 97.8% of male adults released an average of 0.45 ± 0.66 µg of pheromones per hour between 19:00 and 07:00, whereas 93.3% and 88.9% of *M*. *alternatus* released 0.20 ± 1.17 µg and 0.05 ± 0.03 µg per hour during the 07:00–13:00 and 13:00–19:00 periods, respectively (Fig. 7). In contrast, 82.2% and 80% of *M*. *saltuarius* males released 0.151 µg and 0.150 µg per hour during the 13:00–19:00 and 19:00–7:00 periods, with almost no difference between these periods and 91.1% of *M*. *saltuarius* males released a mean of 0.09 µg per hour during 07:00–13:00.

### Mating Behavior of Male Beetles to the Cuticular Extract of Female Beetles

The mating behavior responses of male adults to female extracts are shown in Table 2. For male *M. saltuarius* adults, 17 individuals (85%) exhibited copulatory behavior toward a glass rod coated with extracts from female *M. saltuarius*, while no individuals showed copulatory behavior toward a glass rod coated with extracts from female *M. alternatus*, demonstrating a significant difference (*p* < 0.001). In contrast, for male *M. alternatus* adults, three individuals (15%) exhibited copulatory behavior toward a rod coated with extracts from female *M. alternatus*, and two individuals (10%) toward a rod coated with extracts from female *M. saltuarius*, showing no difference in response to the extracts from females of the two species.

**Table 2:** Copulation rate of male *M*. *alternatus* and *M*. *saltuarius* to female extracts of *M*. *alternatus* and *M*. *saltuarius*

Treatments	Copulation rate (%)	
	M. saltuarius male^1^	M. alternatus male^1^
Female extract of *M*. *salturaius*	85	10
Female extract of *M*. *alternatus*	0	15
	*p* < 0.001^2^	*p* = 1

^1^The numbers of male adults tested with each female extract was 20

^2^The response of males to female extract of the same species versus female extract of a different species was compared using Fisher’s exact test

## Discussion

Many species in the *Monochamus* genus use monochamol as an aggregation pheromone (Pajares et al. 2010, 2013; Fierke et al. 2012; Teale et al. 2011; Lee et al. 2017), which may lead to mate choice errors, especially when species overlap both spatially and temporally. Species such as *Monochamus carolinensis*, *M. galloprovincialis*, and *M. alternatus* can avoid potentially negative heterospecific mating encounters through differences in their geographical distributions (Akbulut and Stamps 2012). In Korea, the distributions of *M*. *alternatus* and *M. saltuarius* were geographically isolated before the PWD outbreak (Kwon et al. 2006), so their common attraction to monochamol has not led to mate choice errors, or interfere with maintaining reproductive isolation. Recent surveys indicate both species have become sympatric in several areas due to the rapid spread of PWD, and increased densities and expanded distributions of both species (Han et al. 2023). Our simultaneous capture of *M*. *alternatus* and *M*. *saltuarius* in pheromone traps in the Gyeongju area confirm the species ovelap in adult activity, both spatialy and temporally.

When geographic ranges of similar species overlap, they may avoid competition by being active in different time of the season (Hanks and Millar 2013; Hanks et al. 2014; Handley et al. 2015; Mitchell et al. 2015). For example, in North America, *Megacyllene caryae* (Gahan) emerges in early spring, while *Megacyllene robiniae* (Förster) emerges in late fall, allowing them to maintain reproductive isolation despite using the same pheromone. We found that *M*. *saltuarius* adults were active about a month earlier than *M*. *alternatus*, but their trap catches overlapped in June and July. When comparing the pheromone release patterns of *M*. *alternatus* and *M*. *saltuarius*, both species consistently release pheromones throughout their lives, and there was no significant difference in the amount of pheromone emitted. Moreover, the two *Monochamus* species continued to release pheromones both before and after mating. Since both species are known to copulate several times with many mates throughout their life span (Fauziah et al. 1987; Li et al. 2021), they are thought to release pheromones continuously both before and after mating. There were subtle differences between the species in the lifetime pattern of pheromone emission. *Monochamus saltuarius* generally began pheromone release approximately 5.6 days earlier than *M*. *alternatus*, but the effect of this on the probability of mate choice error is likely minimal because adults commonly live for weeks and may mate more than once. These results suggest that in the cohabitation area, the overlapping activity periods of the two *Monochamus* beetles and the similarity in lifetime pheromone release patterns are probably insufficient to eliminate mate choice errors.

Longhorn beetles with overlapping seasonal activity periods may divide their active times during the day to maintain reproductive isolation (Millar and Hanks 2017). For example, when *M. scutellatus* and *M. notatus* were collected outdoors, it was reported that they were mainly collected at different times of the day (Skabeikis et al. 2016). This suggests that these two species, which occur in the same region, differentiate their ecological niches by dividing their active times and thereby avoid heterospecific mating (Skabeikis et al. 2016). We showed that *M*. *saltuarius* released more pheromones in the afternoon (13:00–17:00) than did *M*. *alternatus*. In contrast, *M*. *alternatus* released more pheromones from evening to early morning (19:00–07:00) than did *M*. *saltuarius*, although the difference was not significant. Previous studies have reported differences in the daily activity periods of *M. alternatus* and *M. saltuarius* (Nishimura 1973; Kim et al. 2011, 2021). Mating, oviposition, and movement of *M. alternatus* primarily occur at night (Nishimura 1973; Kim et al. 2011) whereas Kim et al. (2021) showed that about 80% of *M*. *saltuarius* were captured in pheromone-baited traps, between 5:00 and 17:00. Based on this evidence, it is possible that differences in the time of day when adults of *M. alternatus* and *M. saltuarius* both emit and respond to pheromone, may help maintain reproductive isolation between them. However, since there is a high chance of overlapping daily activity periods among some individuals within the populations, reproductive isolation could still be subject to interference.

In field assays, the combination of monochamol with *α*-pinene, ethanol, and ipsenol exhibited the strongest attraction effect for *M. saltuarius* (Lee et al. 2017). *Monochamus alternatus*, on the other hand, was more strongly attracted to traps baited with monochamol, *α*-pinene, and ethanol, while ipsenol did not show any synergistic effect with the pheromone blend (Lee et al. 2018). These differences in response to bark beetle aggregation pheromones suggest a potential mechanism by which *M. alternatus* and *M. saltuarius* may reduce interspecific competition in sympatric habitats, in line with the pheromone-free space hypothesis (Rassati et al. 2021).

Male Cerambycidae recognize conspecific female adults through contact with their antennae or maxillary palps and then proceed with mating behavior (Akutsu and Kuboki 1983; Iwabuchi et al. 1987; Kuwahara et al. 1987; Godinez-Aguilar et al. 2009; Ginzel 2003; Millar and Hanks 2017). Previous studies have already reported that male adults of *M. alternatus* and *M. saltuarius* exhibit mating behavior after contact with female adults (Kim et al. 1992, 2006). In this study, 85% of male *M. saltuarius* adults exhibited copulatory behavior toward a glass rod coated with extracts from conspecific females, whereas no individuals showed copulatory behavior in response to extracts from female *M. alternatus*. This indicates that male *M. saltuarius* adults can distinguish conspecifics through substances present on the surface of female bodies. In contrast, only 15% and 10% of male *M. alternatus* adults exhibited copulatory behavior toward glass rods coated with extracts from female *M. alternatus* and *M. saltuarius*, respectively, indicating a low overall copulatory behavior rate with no difference between the extracts from the two species. However, Kim et al. (1992) reported that 55% of Japanese male *M. alternatus* adults exhibited copulatory behavior toward glass rods coated with extracts from female adults, which differs from the results of this study. This difference is likely due to the amount of extract applied; in this study, the extract amount corresponded to a single female, whereas in the Japanese population, the extract amount applied was equivalent to five females, which may account for the variation in mating rates. In any case, the results of this study suggest that *M. alternatus* males showed a lower ability to distinguish conspecific females through substances present on the surface of the female body compared to *M*. *saltuarius* males.

A recent study by Andrade et al. (2025) reported findings similar to those of the present study. Investigating the mechanisms of reproductive isolation between *M. notatus* and *M. scutellatus*, both of which utilize monochamol as a pheromone, they found that *M. scutellatus* tends to emerge earlier in the season and exhibit peak activity during earlier hours of the day. These temporal differences were suggested to contribute in part to reproductive isolation. However, as both species show peak flight activity in the afternoon, temporal segregation alone may not be sufficient to fully prevent reproductive isolation. Andrade et al. (2025) further emphasized that additional mechanisms—such as contact pheromones, host plant preferences, vertical stratification within the forest canopy, and visual cues—may also be involved, and highlighted the need for further research.

In conclusion, our results indicate considerable spatial and temporal ovelap in adult activity of *M. alternatus* and *M. saltuarius*, and that, combined with attraction of both sexes to the same aggregation-sex pheromone, suggests they may be prone to making mate choice errors with potentially negative effects on fitness. We found subtle differences between the species in the timing of initial pheromone release after eclosion and the relative amounts of pheromone emission released during discrete diurnal periods (i.e., 0700–1300, 1300–1900, 1900 − 0700), but we think these are likely insufficient to eliminate heterospecific mating encounters. Our observations of male behavior in response to glass rods coated with cuticular extracts from hetero- and conspecific females suggest that male *M*. *saltuarius* recognize conspecific females via contact sex pheromones are not likely to attempt copulation with female *M*. *alternatus*. However, the same may not be true for male of *M*. *alternatus*, which displayed similar frequencies of attempted copulation with extracts of females of both species. Mate choice errors by male *M*. *alternatus* could have negative consequences on the reproductive success of both species. However, as competition between the two species in cohabitation areas is influenced by various biotic and abiotic factors, further research is required to determine which species may have a survival advantage in cohabitation areas.

## Electronic Supplementary Material

Below is the link to the electronic supplementary material.

Supplementary Material 1

## Data Availability

The datasets generated and/or analyzed during the current study are available from the corresponding author upon reasonable request.
