# Supplementary material for: Does the Difference in the Aggregation-Sex Pheromone Release Pattern Between Monochamus alternatus Hope (Coleoptera: Cerambycidae) and Monochamus saltuarius Gebler Ensure Reproductive Isolation in the Cohabitation Area?
Source: J Chem Ecol. 2025 Jun 6;51(3):65. doi: 10.1007/s10886-025-01617-y (PMC12144045; doi:10.1007/s10886-025-01617-y)
Supplement: Supplementary file 1 — Supplementary Material 1 [file 10886_2025_1617_MOESM1_ESM.pdf]

Table S1. Seasonal flight time of *M. alternatus* and *M. saltuarius* in *Pinus densiflora* forest stands located in Gyeongju City

Month	Survey period	*M. alternatus*			*M. saltuarius*		
		Female	Male	Total	Female	Male	Total
Middle April	April 14 ~ 16	0	0	0	0	0	0
Late April	April 23 ~ 26	0	0	0	1	8	9
Early May	May 6 ~ 8	0	0	0	44	9	53
Middle May	May 18 ~ 20	0	0	0	72	31	103
Late May	May 27 ~ 29	2	0	2	49	25	74
Early June	June 6 ~ 8	76	22	98	28	20	48
Middle June	June 17 ~ 19	150	20	170	12	16	28
Late June	June 26 ~ 28	111	22	133	7	0	7
Early July	July 4 ~ 6	148	31	179	2	0	2
Middle July	July 16 ~ 18	119	17	136	3	0	3
Late July	July 26 ~ 28	55	14	69	1	0	1
Early August	August 5 ~ 7	43	5	48	0	0	0
Middle August	August 18 ~ 20	37	10	47	1	0	1
Late August	August 26 ~ 28	27	8	35	1	0	1
Early September	September 8 ~ 10	9	2	11	0	0	0
Middle September	September 19 ~ 21	22	7	29	0	0	0
Late September	September 28 ~ 30	5	1	6	0	0	0
Early October	October 9 ~ 11	10	2	12	0	0	0
Middle October	October 19 ~ 21	2	1	3	0	0	0
Late October	October 28 ~ 30	0	1	1	0	0	0

Table S2. The daily amount of pheromone emitted per individual *M. alternatus* (MA01-10) after cuticular sclerotization

day	Daily pheromone emission per individual *M. alternatus* (μg)									
	MA01	MA02	MA03	MA04	MA05	MA06	MA07	MA08	MA09	MA10
1	0	0	0	0	0	0	0	0	0	0
2	0		0	0	0	0	0	0	0	0
3	0	0	0	0	0	0	0	0	0	0
4	0	0	0	0	0	0	0	0	0	0
5	0	0	0	0	0	0	1.1	0	0	0
6	0	0	0	0	3.0	0	0	7.9	0	0
7	0	0	0	0	3.1	0	4.1	6.2	5.0	0
8	0	0	0	0	9.2	0	4.9	10.1	5.9	0
9	0	0	0	0	0	7.6	6.6	12.4	0	0
10	0	0	0	0	12.1	8.6	12.4	4.9	7.1	0
11	0	0	0	0	6.1	0	0	7.0	4.9	0
12	11.2	0	0	0	8.8	14.4	0	7.1	6.7	6.3
13	7.0	0	0	0	5.5	30.6	10.3	20.6	4.7	6.0
14	7.5	0	0	0	8.3	4.3	0	2.5	7.4	0
15	0	0	0	0	0	0	12.5	2.3	4.6	5.3
16	11.6	0	0	5.1	1.2	8.5	6.0	4.2	1.2	4.8
17	8.7	0.1	10.9	0	4.9	4.8	1.8	9.9	10.3	4.3
18	13.7	0	0	0	1.1	8.1	10	3.6	9.9	6.8
19	3.4	0.1	7.3	12.6	5.0	7.2	10.2	7.9	6.7	5.5
20	7.3	5.0	6.8	0	0	9.2	9.5	4.7	4.5	5.2
21	6.2	0.1	5.3	2.9	1.5	5.3	9.5	10.7	1.2	1.4
22	16.9	0.1	5.2	0	3.4	7.4	11.8	4.4	7.4	2.7
23	4.2	0.1	2.7	4.6	0	8.7	5.0	0	0	5.1
24	14.6	0.8	5.1	8.8	2.1	3.1	13.1	4.2	0	3.8
25	4.4	2.9	4.0	0	3.9	4.5	14.0	9.6	4.1	7.5
26	10.6	5.3	1.1	2.9	0	2.8	4.3	7.3	2.3	0
27	2.6	1.1	0.9	0	6.8	1.1	6.2	2.7	1.9	12.2
28	3.0	3.0	1.1	2.8	0	0	3.0	0	0	3.5
29	1.0	1.5	3.2	0.6	0	0	9.9	2.7	3.5	5.9
30	15.4	2.3	4.8	13.4	3.1	4.9	4.8	4.4	0	0
31	0.4	0	0	0	6.2	7.8	3.7	15.8	0	4.1
32	10	0	4.2	11.1	0	1.8	2.1	0	0	0
33	1.5	0	5.9	0	0.4	1.4	6.7	4.6	3.0	2.2
34	6.4	1.4	3.0	0.9	3.6	8.7	8.9	4.3	1.2	5.8

35	4.3	1.0	15.9	9.6	3.0	5.9	6.4	12.6	1.8	3.1
36	10.4	0	8.8	0	4.4	7.8	4.6	3.2	1.5	1.0
37	5.5	0	7.0	3.8	3.9	10.4	7.2	3.3	2.6	0
38	2.3	0	0.8	0	11.5	3.6	5.3	4.7	7.8	0
39	1.0	0	6.7	0.6	0.1	2.3	10.5	5.1	3.6	7.7
40	7.4	0	3.2	0	0.3	11.2	6.1	3.4	0.9	6.5
41	3.0	1.0	9.0	0	2.9	9.3	5.5	7.4	10.1	0.9
42	5.6	0	1.2	4.0	7.2	6.2	5.7	3.3	0	3.2
43	5.6	0	0	2.2	0	2.8	3.9	9.6	0	3.4
44	0.6	0	0	0.7	5.4	3.5	2.4	2.1	1.5	6.2
45	0	0	0	0	1.5	4.7	6.3	6.8	3.6	7.3
46	0	0	0	0	8.7	1.8	4.8	5.5	0	4.1
47	0	Death	Death	0	1.2	5.5	3.4	4.9	0	4.6
48	0.3	-	-	0	1.6	2.4	3.5	1.3	3.8	1.2
49	0	-	-	1.3	2.1	4.0	2.3	5.9	6.1	1.5
50	0	-	-	0	0	1.2	0	4.6	2.1	0
51	0.6	-	-	Death	0	0	2.6	4.4	2.4	1.5
52	Death	-	-	-	0	0	0.5	1.8	1.2	6.0
53	-	-	-	-	0	0	2.0	0	5.1	4.4
54	-	-	-	-	0	2.0	1.1	2.5	0	3.2
55	-	-	-	-	0	1.4	0	1.9	3.5	1.4
56	-	-	-	-	0.8	0	0	1.8	0	1.7
57	-	-	-	-	0	0.5	2.4	0	0	0
58	-	-	-	-	0.5	0	0.9	0	0	0
59	-	-	-	-	0	0.3	0.2	0	0	0
60	-	-	-	-	0	0	0	0.7	0	0
61	-	-	-	-	0.2	0.6	1.3	0	0.5	0
62	-	-	-	-	0	0	0	1.5	0.6	2.8
63	-	-	-	-	0	4.5	1.5	2.8	0	0
64	-	-	-	-	0	0.6	0.6	0	0	2.5
65	-	-	-	-	0	0	0	10.2	0	0.9
66	-	-	-	-	0.4	1.3	1.5	4.8	0	0.6
67	-	-	-	-	2.4	8.5	0.6	6.1	5.6	1.5
68	-	-	-	-	0	0	0	0.1	0	0.4
69	-	-	-	-	0	0	0	1.0	1.7	0
70	-	-	-	-	0	0	0	0	0	0
71	-	-	-	-	0	0	0	0	0	0
72	-	-	-	-	0	0	0	0	0	0
73	-	-	-	-	0	4.6	2.9	0	0	0
74	-	-	-	-	0	0	0	0	0	0

75	-	-	-	-	0	0	0	0	0	0
76	-	-	-	-	0	0	0	0	0	0
77	-	-	-	-	0	0.1	0	0	0	0
78	-	-	-	-	0	0	Death	0	0	0
79	-	-	-	-	0	0	-	0	0	0
80	-	-	-	-	0	0	-	0	Death	0
81	-	-	-	-	0	0	-	0	-	0
82	-	-	-	-	0	0	-	0	-	0
83	-	-	-	-	0	0	-	0	-	0
84	-	-	-	-	0	0	-	0	-	0
85	-	-	-	-	0	0	-	0	-	0
86	-	-	-	-	0	0	-	0	-	0
87	-	-	-	-	0	0	-	0	-	0
88	-	-	-	-	0	0	-	0	-	0
89	-	-	-	-	0.5	0.5	-	0	-	0
90	-	-	-	-	0	0.6	-	0	-	0
91	-	-	-	-	0.5	0	-	0	-	0.4
92	-	-	-	-	0	0	-	0	-	0
93	-	-	-	-	0.4	0	-	0	-	0
94	-	-	-	-	0	0	-	Death	-	0
95	-	-	-	-	0	0	-	-	-	1.4

Table S3. The daily amount of pheromone emitted per individual *M. saltuarius* (MS01-12) after sclerotization

Day	Daily pheromone emission per individual *M. saltuarius* (μg)											
	MS01	MS02	MS03	MS04	MS05	MS06	MS07	MS08	MS09	MS10	MS11	MS12
1	0	0	0	1.5	0	0	0	0	0	0	0	0
2	0.5	0	0	0.8	0	0	0	2.2	0	0	0	0
3	0	0	0	0	0	0	0	0	0	0	0	0
4	1.1	9.1	0	11.6	0	6.6	0	4.1	1.4	0	0	0
5	0	1.5	3.3	7.6	4.8	0	0	0	0	0	0	0
6	0	0	3.4	0	0	1.3	1.0	0.9	0	0	0	0
7	0.6	0	1.3	0	0	0	0	2.6	0	0	0	1.0
8	12.4	0	0	6.5	13.3	0	1.5	0.7	4.1	0	0	4.9
9	9.6	0	5.1	1.8	12.1	3.3	7.1	5.0	20.3	0	0	4.1
10	0	1.8	2.0	1.3	1.6	1.4	6.1	1.0	1.6	1.4	3.8	4.7
11	8.6	8.7	0	2.6	0	0.4	4.8	2.8	4.0	0.8	1.3	1.0
12	0	0	0	0	0	0	1.3	1.6	1.8	1.0	3.4	0
13	1.1	0.6	1.3	0	2.7	1.2	1.4	2.7	1.5	0.7	3.5	0.7
14	10	0	3.1	0	7.8	1.7	0	2.2	1.6	6.9	1.9	0
15	1.1	0	0.9	0	0.4	1.8	0	5.3	2.1	13.2	4.1	1.9
16	0	0	0.3	0	0.6	0.3	1.7	1.8	1.9	0	12.1	8.6
17	25.6	10.8	2.8	0	0	28.2	1.7	1.6	7.9	0	0	2.3
18	36.2	5.5	2.5	0.9	2.9	13.3	2.3	2.8	9.0	2.3	6.2	0
19	2.4	2.2	3.3	0.9	0.3	14.7	1.9	4.0	2.5	7.3	3.0	2.3
20	0	2.8	0.4	0	0.2	2.5	1.9	2.2	12.7	0	6.6	9.7
21	4.7	0	0	0	0	3.0	1.7	3.0	2.6	13.9	2.4	0
22	0	0	0	0	0	0	0	2.0	2.7	2.2	8.3	3.7
23	1.9	0	0	0	0	0	0	2.1	5.4	1.8	12.3	5.7
24	2.4	1.3	0	7.3	0	1.7	3.6	7.3	3.1	12.5	8.5	3.8
25	0	0	0	0	2.3	5.0	0	3.2	5.1	4.9	0	0
26	0	0	0	0	0	0	1.6	4.0	13.9	3.1	7.5	9.2
27	0	0	0	0	0	0	0	1.2	1.9	1.1	1.1	1.4
28	0	0	0	0	0	0	1.2	6.9	1.2	0.9	1.7	1.0
29	0	0	0	0	3.8	1.7	0.9	0	1.2	0.9	0.9	0.9
30	0	0	0	0	0	0	2.2	1.3	1.7	0	2.2	0.9
31	0	0	0	0	7.4	0	0	0.9	0.9	0.9	1.0	0
32	0	0	0	0	0	0	0	0	1.0	0	0.8	0
33	0	0	0	0	0	0	0	2.7	6.4	0.7	6.3	9.2
34	0	0	0	0	0	0	1.6	0.8	1.1	0	3.4	0.8

35	0	0	0	0	0	0	0.6	0	0.6	0.6	1.0	0.5
36	0	0	0	0	6.9	1.7	0.8	0	1.7	5.8	14.5	13.9
37	0	0	0	0	0	0	1.2	5.8	17.6	1.3	16.1	1.7
38	0	0	0	0	0	0	1.2	0.6	4.0	0	2.9	2.6
39	0	0	0	0	0	0	0.1	12.7	2.2	0	8.4	5.8
40	0	0	0	0	0	0	0	0	0	0	74.9	13.2
41	0	0	0	0	0	0	0	0	0	1.7	2.8	2.0
42	0	0	0	0	0	0	0	1.4	2.4	0	1.3	7.8
43	0	0	0	0	0	0	0	0	0	0	0	0.2
44	0	0	0	0	2.3	0	0	0	0	2.1	0	0
45	0	0	0	0	0	0	0	0	0	0	1.5	0.7
46	Death	10.7	2.3	0	0	0	0	0	3.0	2.6	1.4	1.8
47	-	0	0	0	0	0	0	Death	5.0	0	2.2	0
48	-	0	0	0	16.7	0	1.4	-	1.8	0	1.9	0
49	-	0	3.2	0	4.7	2.3	4.4	-	3.9	0	10.9	3.0
50	-	0	0	0	0	0	2.4	-	2.1	0	10.6	5.9
51	-	0	0	0	0	0	2.7	-	3.0	3.6	5.0	1.7
52	-	0	0	0	0	0	1.6	-	2.9	6.0	1.6	0
53	-	0	0	0	0	0	1.5	-	0	1.9	0	0
54	-	0	0	0	2.3	0	7.9	-	2.1	0	Death	0
55	-	0	4.4	4.8	0	3.2	0	-	1.6	0.2	-	8.7
56	-	0	3.2	5.4	0	1.4	1.1	-	0.9	5.9	-	9.6
57	-	0	0	0	Death	0	1.2	-	1.1	1.5	-	2.1
58	-	0	7.1	0	-	0	14.3	-	1.3	15.4	-	0.1
59	-	0	0	0	-	0	3.1	-	1.2	5.3	-	1.2
60	-	0	0	0	-	0	1.1	-	1.1	1.7	-	0
61	-	0	0	0	-	0	1.1	-	1.1	8.2	-	1.1
62	-	0	0	0	-	0	1.1	-	0	4.4	-	1.1
63	-	0	0	0	-	0	1.1	-	1.1	4.1	-	1.1
64	-	0	0	0	-	0	1.4	-	1.1	2.6	-	1.3
65	-	0	3.1	0	-	0	1.7	-	1.5	7.3	-	1.3
66	-	0	0	0	-	0	1.7	-	3.2	5.0	-	1.1
67	-	0	0	0	-	Death	1.2	-	2.0	9.0	-	1.1
68	-	0	0	0	-	-	1.5	-	0.6	7.1	-	0.2
69	-	0	0	0	-	-	5.2	-	8.8	7.2	-	4.0
70	-	0	0	0	-	-	0.8	-	0.2	1.3	-	1.2
71	-	0	0	0	-	-	3.1	-	2.3	2.2	-	0.4

72	-	0	0	6.5	-	-	1.6	-	1.7	4.2	-	0.3
73	-	0	0	Death	-	-	0.4	-	0.9	4.9	-	1.0
74	-	2.7	0	-	-	-	0.9	-	1.2	1.3	-	1.7
75	-	0	0	-	-	-	0	-	0.9	0.8	-	0.8
76	-	0	0	-	-	-	1.0	-	0.8	1.1	-	0.8
77	-	2.5	10.8	-	-	-	0	-	0	0	-	0.8
78	-	2.8	0	-	-	-	0	-	0	0	-	1.1
79	-	0	0	-	-	-	0.7	-	0	5.3	-	0.7
80	-	2.4	0	-	-	-	0	-	0	0	-	0
81	-	0	0	-	-	-	0	-	0	1.2	-	0.3
82	-	0	0	-	-	-	0.1	-	0	0.8	-	0.1
83	-	0	0	-	-	-	0	-	0.1	0.1	-	0.1
84	-	0	0	-	-	-	0	-	Death	0	-	0.7
85	-	0	Death	-	-	-	0.8	-	-	0.9	-	0.9
86	-	0	-	-	-	-	0.7	-	-	9.1	-	0.7
87	-	0	-	-	-	-	0.8	-	-	1.3	-	0.7
88	-	0	-	-	-	-	0.1	-	-	8.2	-	0.1
89	-	0	-	-	-	-	0.1	-	-	0.8	-	0.2
90	-	0	-	-	-	-	Death	-	-	18.8	-	0.1
91	-	0	-	-	-	-	-	-	-	2.1	-	1.8
92	-	0	-	-	-	-	-	-	-	2.1	-	1.9
93	-	0	-	-	-	-	-	-	-	8.7	-	1.8
94	-	0	-	-	-	-	-	-	-	8.8	-	1.8
95	-	0	-	-	-	-	-	-	-	0	-	0
96	-	0	-	-	-	-	-	-	-	6.3	-	2.0
97	-	0	-	-	-	-	-	-	-	3.0	-	1.8
98	-	0	-	-	-	-	-	-	-	2.8	-	1.8
99	-	2.1	-	-	-	-	-	-	-	1.8	-	1.9
100	-	0	-	-	-	-	-	-	-	Death	-	1.8

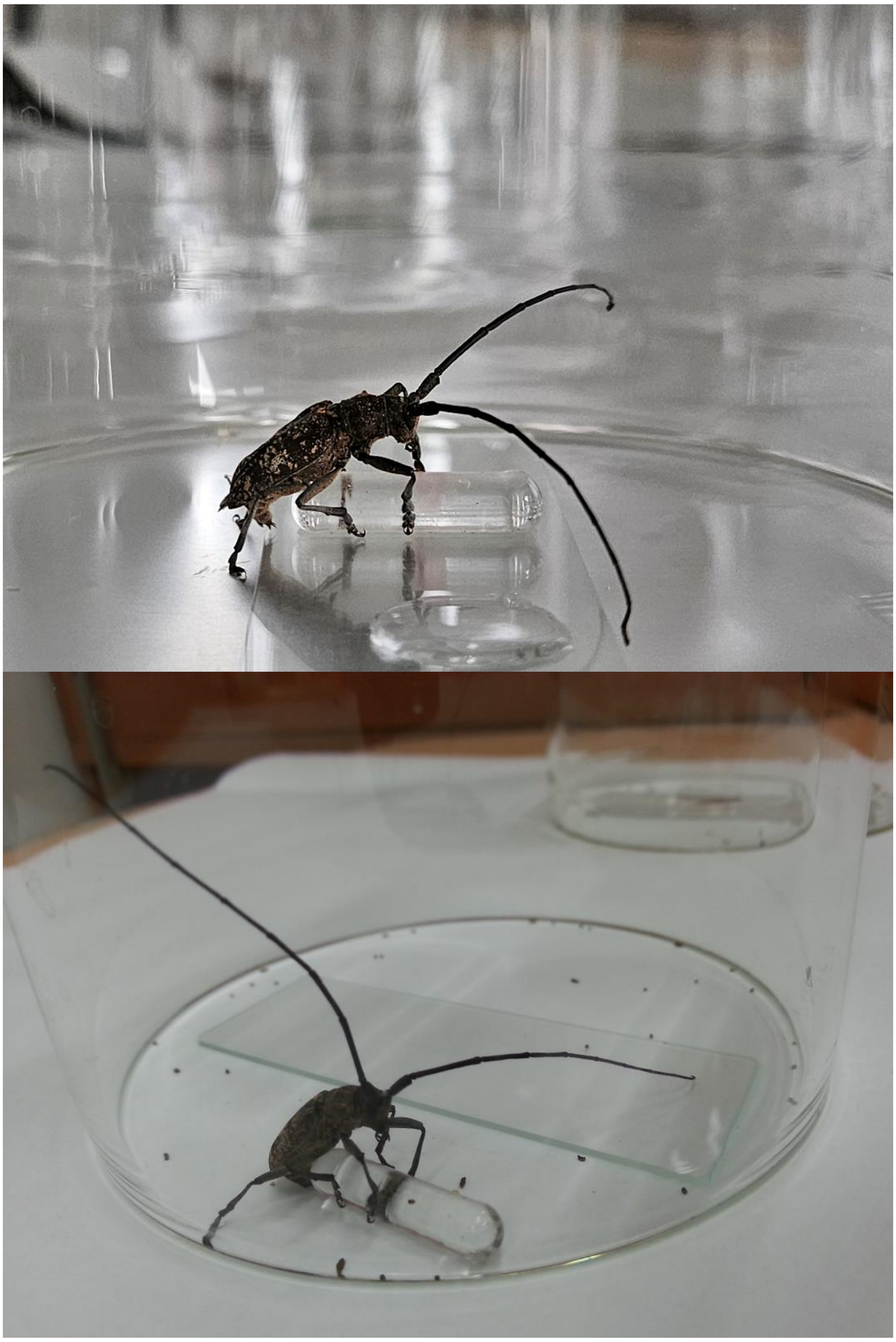

Fig S1. Mating behavior of male *M. saltuarius* (upper) and *M. alternatus* (down) to glass rods baited with female extracts
